# Understanding the Adaptive Mechanisms of Plants to Enhance Phosphorus Use Efficiency on Podzolic Soils in Boreal Agroecosystems

**DOI:** 10.3389/fpls.2022.804058

**Published:** 2022-03-15

**Authors:** Muhammad Nadeem, Jiaxu Wu, Hamideh Ghaffari, Amana Jemal Kedir, Shamila Saleem, Alain Mollier, Jaswinder Singh, Mumtaz Cheema

**Affiliations:** ^1^School of Science and the Environment, Memorial University of Newfoundland, Corner Brook, NL, Canada; ^2^Department of Agronomy, Shahrekord University, Shahrekord, Iran; ^3^Environmental Science Program, Memorial University of Newfoundland, St. John's, NL, Canada; ^4^Department of Agriculture Extension, Government of Punjab, Khanewal, Pakistan; ^5^INRAE, UMR 1391 ISPA, Bordeaux Science Agro, Villenave d'Ornon, France; ^6^Department of Plant Science, McGill University, Ste-Anne-de-Bellevue, QC, Canada

**Keywords:** acidic soils, nutrient use efficiency, podzol, P transporters, root architecture, seed P reserves, short growth seasons, sustainable agroecosystem

## Abstract

Being a macronutrient, phosphorus (P) is the backbone to complete the growth cycle of plants. However, because of low mobility and high fixation, P becomes the least available nutrient in podzolic soils; hence, enhancing phosphorus use efficiency (PUE) can play an important role in different cropping systems/crop production practices to meet ever-increasing demands in food, fiber, and fuel. Additionally, the rapidly decreasing mineral phosphate rocks/stocks forced to explore alternative resources and methods to enhance PUE either through improved seed P reserves and their remobilization, P acquisition efficiency (PAE), or plant's internal P utilization efficiency (IPUE) or both for sustainable P management strategies. The objective of this review article is to explore and document important domains to enhance PUE in crop plants grown on Podzol in a boreal agroecosystem. We have discussed P availabilities in podzolic soils, root architecture and morphology, root exudates, phosphate transporters and their role in P uptake, different contributors to enhance PAE and IPUE, and strategies to improve plant PUE in crops grown on podzolic soils deficient in P and acidic in nature.

## Introduction

Phosphorus (P) is a critical and important mineral required by growing plants to sustain their growth and productivity (Malhotra et al., 2018). It is a fundamental player in various biochemical processes occurring in growing plants (White et al., 2008; Malhotra et al., 2018) and is, therefore, considered as the backbone of seed germination, seedling establishment, and plant growth, development, and reproduction. Thus, it is necessary to complete the life cycle of plants. Heterotrophic seed P is the primary source to support seedling establishment during the onset of germination and the first few weeks of early growth (Nadeem et al., 2011). For onward plant growth and development, P is taken by root systems from soils. However, because of low P mobility and availability in soils, substantial amounts of autotrophic P (soil P) must be supplied from organic or inorganic fertilizer sources (Grant and Flaten, 2019). A major source of inorganic P is fertilizers; therefore, farmlands receive significant quantities of P fertilizers each year because of low soil P status worldwide (Lu and Tian, 2017; Ros et al., 2020). However, the applied P becomes unavailable because of higher affinities toward cations and soil organic matter (SOM), rendering it the least mobile and phytoavailable nutrient (Hinsinger, 2001), particularly, in podzolic soils that are rich in aluminum (Al) and iron (Fe) minerals that fix available P pools (Kedir et al., 2021).

Generally, podzols have a coarse sandy texture, shallow depth, acidic pH, and low fertility, which restricts crop growth and productivity (DeBruyn et al., 2011; Ali et al., 2019b). Higher applications of P fertilizers are, therefore, critical to enhancing crop production, but there are alarming concerns on long-term environmentally sustainable use of nonrenewable P resource (Ros et al., 2020). Despite high P applications, PUE is only 15–20% in agricultural croplands, indicating that most of the applied P is not accessible for plant use (Malhotra et al., 2018). It is, therefore, important to review knowledge gaps to enhance PUE on podzolic soils in managed boreal agroecosystems. Therefore, improving soil P availabilities, reducing P fixation, and enhancing P uptake and internal plant P utilization would improve our understating to enhance overall PUE on podzolic soils. Hence, exploring different plant adaptive strategies to enhance PUE is crucial to sustaining agricultural crop productivity and nonrenewable P reserves management to meet growing food and feed requirements (Ramaekers et al., 2010). This review covers three objectives: first, we give an overview of P availabilities in podzolic soils and different factors governing the dynamics of P. Second, we discuss plants' adaptive approaches in P acquisition in podzolic soils. Furthermore, our focus is to explore the contributions of P acquisition and P utilization in enhancing PUE in growing plants. Finally, we suggest potential strategies and interventions to improve crop P uptake and PUE on podzols in boreal agroecosystems for agricultural crop production and maintenance of high farm outputs.

## Phosphorus in Podzolic Soils

### Phosphorus Stocks and Field Applications

Globally, phosphate rock is the main source of phosphatic fertilizer (Cordell et al., 2009). Approximately 90% of all mined phosphate rock is used for agricultural crop production (Nesme et al., 2018). The current global supply of phosphatic fertilizer is 50 MT and is projected to increase to 90 MT by 2050 (FAO, 2019; Nedelciu et al., 2020). Expansion of fertilizer usage in agriculture will boost food production; however, high consumption of nonrenewable P resources will put more pressure on already depleting global P reserves (Ros et al., 2020). Although precision agriculture practices promote the optimum utilization of P resources, only 30% of applied P is used by crops to complete their growth cycle, and 70% of applied P is fixed or released to the environment. Such P losses have significant detrimental effects on ecosystem function highlighted in the last few decades such as reduced water quality and ecological disturbances (Weihrauch and Opp, 2018).

Phosphorus (P) is a complex nutrient existing in organic or inorganic forms in soils (Requejo and Eichler-Löbermann, 2014). Based on soil types and management practices, P is the least phytoavailable nutrient in soils because of its higher fixation capacities by reactive minerals like aluminum (Al^3+^), iron (Fe^2+^), magnesium (Mg^2+^), calcium (Ca^2+^), and organic compounds, which slow its diffusion rate in plant root rhizospheres (Vance et al., 2003). Phytoavailable P species in a soil solution either exist in the form of primary orthophosphate (${\text{H}}_{\text{2}}{\text{PO}}_{\text{4}}^{\text{ −}}$) or secondary orthophosphate (${\text{HPO}}_{4}^{2-}$) anions depending on soil pH. Primary orthophosphate is the dominant form of podzols common in boreal agroecosystems. The phytoavailability of organic P forms in soil is less studied and depends on microbial activities in the rhizosphere and on environmental factors. P concentrations in a soil solution are typically lower than 10 μM because of P's high fixation and low solubility in acidic and calcareous soils (Bieleski, 1973), leaving significant global croplands (70%) P-deficient (von Uexküll and Mutert, 1995). The podzol rich in Al^3+^ and Fe^2+^ minerals has high P retention capacity and, consequently, lower P availability (Peltovuori, 2007). Mineral P fertilizers are the principal input to enhance P availability in modern agricultural production systems; however, the applied P is rapidly immobilized by SOM and minerals in acidic soils (Hinsinger, 2001). Hence, the supplemented P through fertilizers or inherent soil P becomes unavailable for plant utilization unless hydrolyzed to release P in plant rhizosphere with various root exudates or active soil microbial communities (Alori et al., 2017; Ali et al., 2019b). Therefore, efficient utilization of P resources is a recent pressing question regarding sustainable crop production, better economic return, human wellbeing, and environmental sustainability. Therefore, it is crucial to explore advanced strategies and agronomic management to enhance plant P uptake and internal P translocation and remobilization to develop P-efficient genotypes in boreal agroecosystems to effectively utilize P resources and improve crop production.

Based on the dose-response, high fertilizer input has been thought to obtain a high crop yield. However, the growing concern on depletion of P reserves and environmental pollution created opportunities to explore the efficient utilization of P in the agriculture industry (Cordell and White, 2013). A meta-analysis study documented that overuse of P would not improve crop yield (Ros et al., 2020); instead, it causes aquatic pollution. For example, a field experiment on podzolic soil revealed that 22 kg P ha^−1^ resulted in a higher carrot yield than 110 kg P ha^−1^ (Sanderson and Sanderson, 2006). Thus, it is essential to understand P availability in podzolic soils, and utilization efficiency by plants might help reduce environmental P buildup and losses for economic and environmental benefits.

### Factors Affecting P Availabilities in Podzolic Soils

Generally, the availability of P in mineral soils like Podzol depends on its buffering capacity, mineral and organic matter content, management history, and environmental factors (Quintero et al., 2003; Pierzynski et al., 2005; Kang et al., 2009). Unique characteristics of podzolic soils, such as increased acidity, higher exchangeable Al^3+^ and Fe^2+^ content, lower cation exchange, and water retention capacity result in higher P retention capacity and low soil P concentration (<0.01 mg P L^−1^), typical in boreal agroecosystems (Pierzynski et al., 2005; Paul et al., 2011). Under natural conditions, the podzolization process facilitates leaching of cations such as Al^3+^, Fe^2+^, and Ca^2+^, dissolved organic matter, and chelates from organic layers or eluviated profiles that accumulated in illuviated profiles (Paul et al., 2011; Grand and Lavkulich, 2013; Krettek and Rennert, 2021). However, most agricultural podzolic soils have lost the organic and eluviated profile or a small proportion is mixed with illuviated B horizon rich in Al and Fe minerals during initial or successive soil preparation (Paul et al., 2011). Mainly in converted podzol, different forms of Al species (exchangeable, charged organometallic and poorly crystalline surfaces, aluminosilicates, and oxyhydroxides) and charged soil organic matters may be responsible for fixing or retaining plant-available P in soil solutions (Grand and Lavkulich, 2013). Thus, based on soil pH, mineral soils act as P sink until buffering capacity is satisfied. For example, long-term managed orthic humo-ferric podzol developed on a glacial till, with 10% organic matter (loss-on-ignition), Melich-3 Al^3+^ to Fe^2+^ ratio of 8:3, and P of 57 mg L^−1^ had higher P retention capacity (Kedir et al., 2021). Also, there might be counter effects of long-term liming application to suppress acidity and Al^3+^ toxicity, which could lead to calcium phosphate precipitation in long-term managed soils with near neutral pH, supported with high (2,155 mg L^−1^) Ca^2+^ content compared to 41 mg L^−1^ in recently converted podzolic soils in a boreal agroecosystem (Kedir et al., 2021). Grand and Lavkulich (2013) reported that in most acidic soils, the highest proportion of P is firmly fixed by Al^3+^-based amorphous minerals or organic Al^3+^ materials. Hence, the intensity of Al^3+^ in acidic soils might reflect P dynamics based on pH that can be used as an indicator to improve P availability for plant uptake.

Additionally, soil management methods such as tillage, liming, manure applications, and the shallow nature of soils could affect P availability in podzolic soils (Pierzynski et al., 2005; Shen et al., 2011). Plant root morphology and physiology also play a crucial role in plant growth by recycling P in the rhizosphere (Fageria et al., 2014). The rooting depth of different plant species varies and can be affected by soil compaction, adaptability, nutrient availability, and soil characteristics such as moisture, temperature, pH, and mineral horizon (Eh) for redox potential (Fageria et al., 2014). Furthermore, P uptake by plant roots may be influenced by genotypes, root architecture, length, hair, density and structure, and the rhizobium (Shen et al., 2011; Fageria et al., 2013). Root exudates, mainly organic acids (citric acid, malic acid, and oxalic acids), might solubilize and mobilize fixed P forms (Tawaraya et al., 2013), but this requires further investigation in podzolic soils to improve P acquisition efficiency. Therefore, it is important to understand all factors affecting P availability to enhance PUE in growing plants on podzolic soils in boreal agroecosystems.

### Importance of Soil P Tests to Enhance PUE

The first step in enhancing PUE is to assess accurately the P status of soil by proper soil P testing. About a dozen P-tests was developed to quantify plant-available P in the soils depending on the soil types and properties, specifically soil pH (Table 1). It is important to choose and calibrate proper soil P test to assess plant-available P in podzolic soils. Different agri-environmental P tests targeted different P pools (Table 1); water and resin extracted readily available dissolved P and represented instantaneous P supply to roots *via* mass flow effect (Nafiu, 2006; Wang et al., 2015). Generally, diluted acid or base solutions are used to extract different P pools in soils. For example, acidic solutions of the Bray method (Bray and Kurtz, 1945), Mehlich (Mehlich, 1984), and citric acid (Thompson, 1995) can extract easily soluble and plant-available P forms as an indicator of P fertility status (Jones, 2001). These methods are designed for podzolic soils rich in Al^3+^ and Fe^2+^ minerals and slightly neutral soils (Ketterings and Flock, 2005) that are affected by soil management and environmental factors (Messiga et al., 2015). Most P tests only target to measure P pools, while potentially soluble and available organic P is not correctly accounted for in P recommendation (Ziadi et al., 2013). For example, the Mehlich-3 test performed in most North American laboratories only targets to measure P and multiple cations relevant to plant nutrition (Ziadi et al., 2009). On the other hand, specific P tests developed for particular soil types or regions might over- or underestimate P when applied on different soil types (Nafiu, 2006). Kedir et al. (2021) evaluated P extractability in podzolic soils by nine P-tests following the order water ≤ Morgan ≤ ammonium bicarbonate-diethylenetriaminepentaacetic acid (AB-DTPA) < Mehlich-1 ≤ Bray-1 ≤ Mehlich-3 ≤ Olsen < Bray-2 < citric acid. Similarly, Zehetner et al. (2018) reported weaker extraction methods to correlate better while grouping based on pH. Thus, proper selection and calibration of P tests against P uptake or crop yield under local environmental and field management conditions help to improve plant P use efficiency in podzolic soils. Additionally, more than one P test might be beneficial to measure plant-relevant P pools in podzolic soils.

**Table 1:** Selection of proper soil phosphorus test based on soil properties and targeted phosphorus species (Elrashidi, 2010; Zehetner et al., 2018; Kedir et al., 2021).

**Soil**	**pH**	**Minerals**	***P*-tests**
Acidic	<6.0	Al-P, Fe-P, Mn-P, and organic P	Bray-I, Mehlich-1, Mehlich-3, citric acid, Morgan, water extraction, calcium chloride, Iron oxide impregnated paper test, and anion exchange resin
Slightly acid to slightly alkaline	6.0–7.2	Al-P, Fe-P, Mn-P, Mg-P, Ca-P, and organic P	Bray-I, Bray-II, Mehlich-1, Mehlich-3, citric acid, Morgan, ammoniumbicarbonate diethylenetriaminepentaacetic acid (AB-DTPA), Olsen, water extraction, calcium chloride, Iron oxide impregnated paper test, and anion exchange resin
Calcareous	>7.2	Ca-P, Mg-P, and organic P	AB-DTPA, Olsen, water extraction, calcium chloride, Iron oxide impregnated paper test, and anion exchange resin

## Phosphorus Use Efficiency

**Figure 1:**
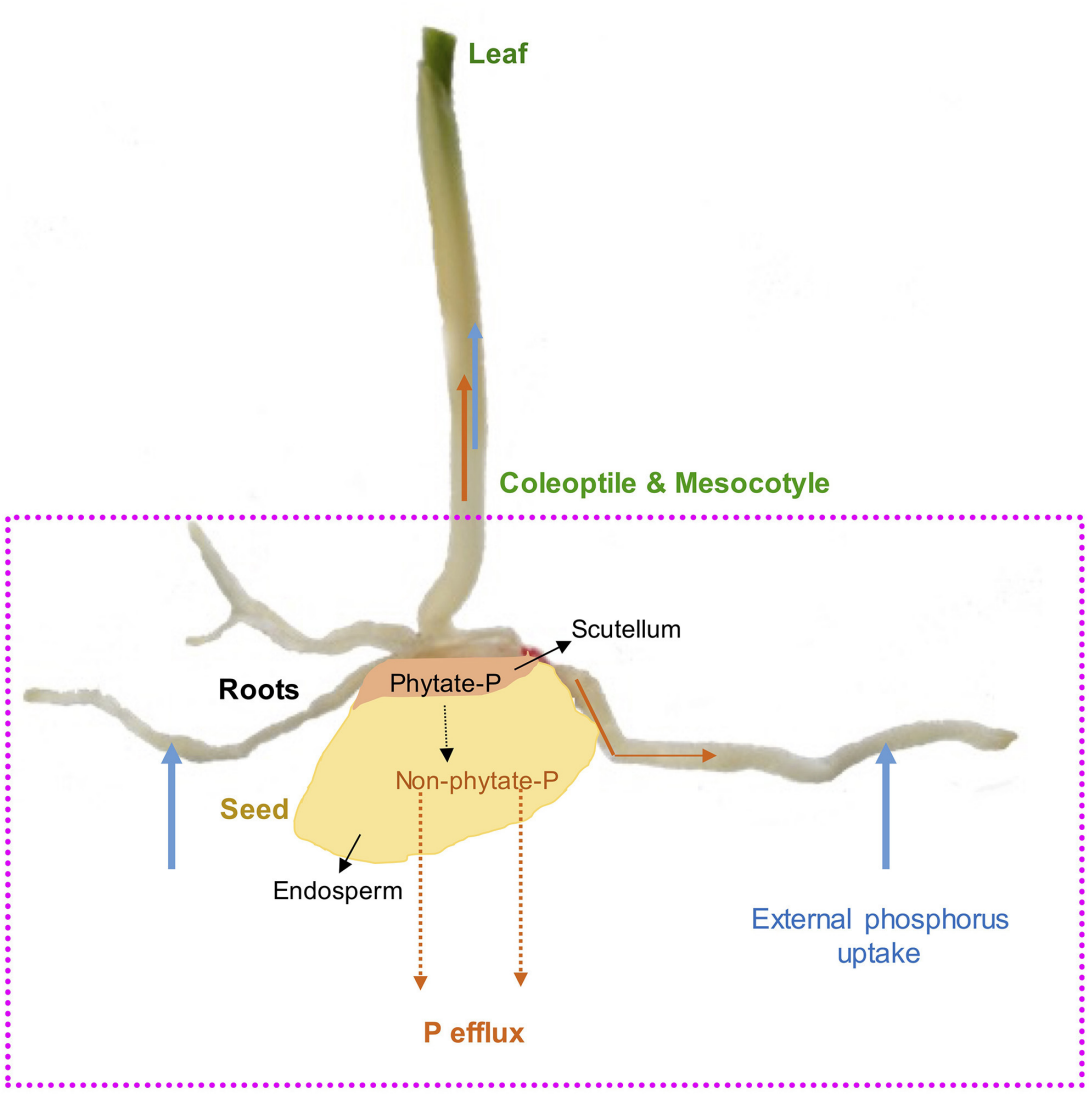
Maize (*Zea mays L*.) as a model seed showing endosperm and scutellum. Seed phytate-P hydrolysis during germination, translocations to developing roots, coleoptile, mesocotyle, and leaves as well as external P uptake and P efflux during germination and early growth stages.

**Figure 2:**
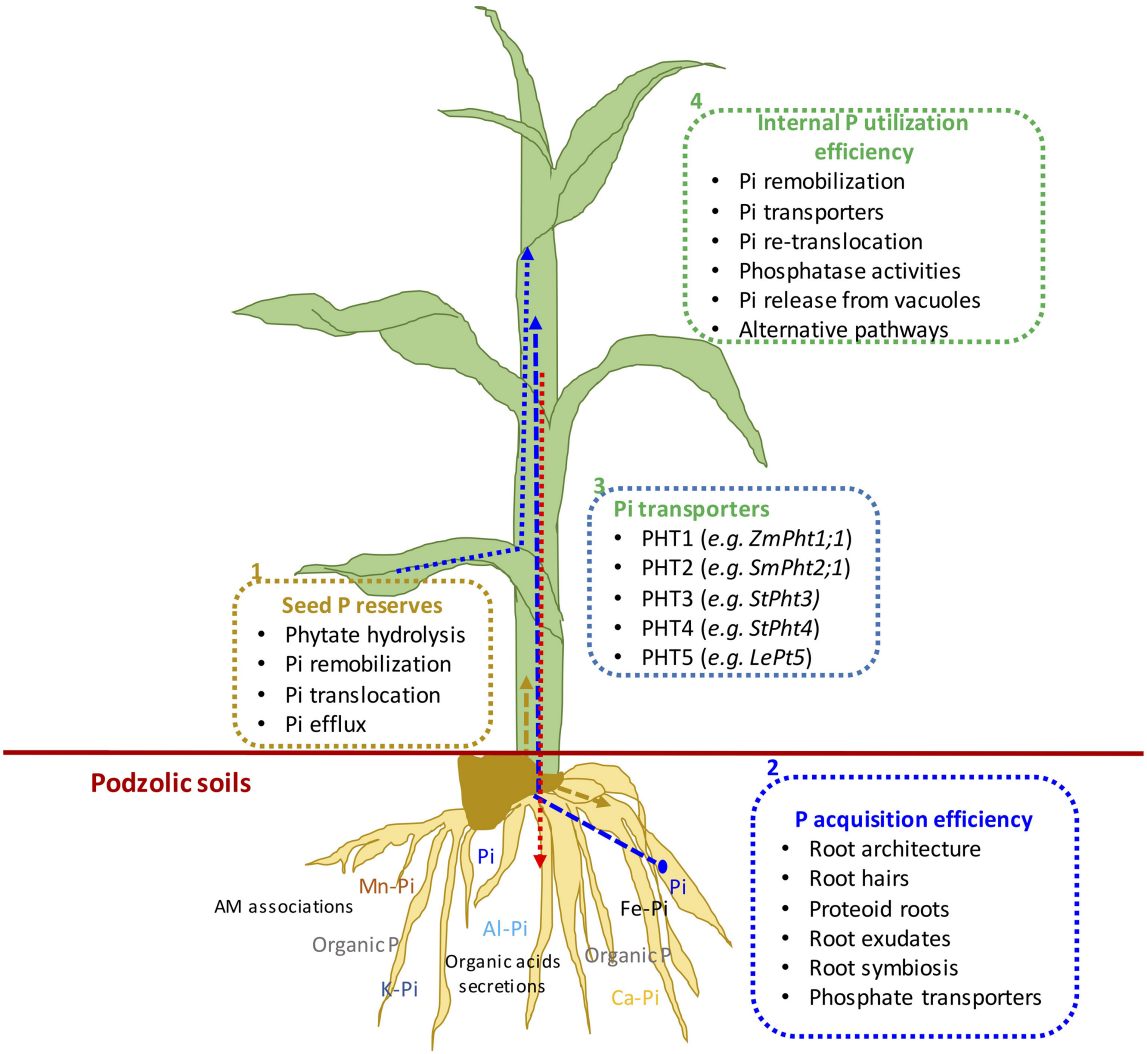
(1) Schematic representation of mechanisms of seed phosphorus remobilization, (2) P acquisition efficiency, (3) P transporters, and (4) internal P utilization efficiency from germination to final plant harvest in maize plant. Dotted arrows show different P fluxes in growing seedlings: Blue represents external P uptake, gold represents remobilized seed P, and pink arrow shows P translocation from leaves to roots.

**Figure 3:**
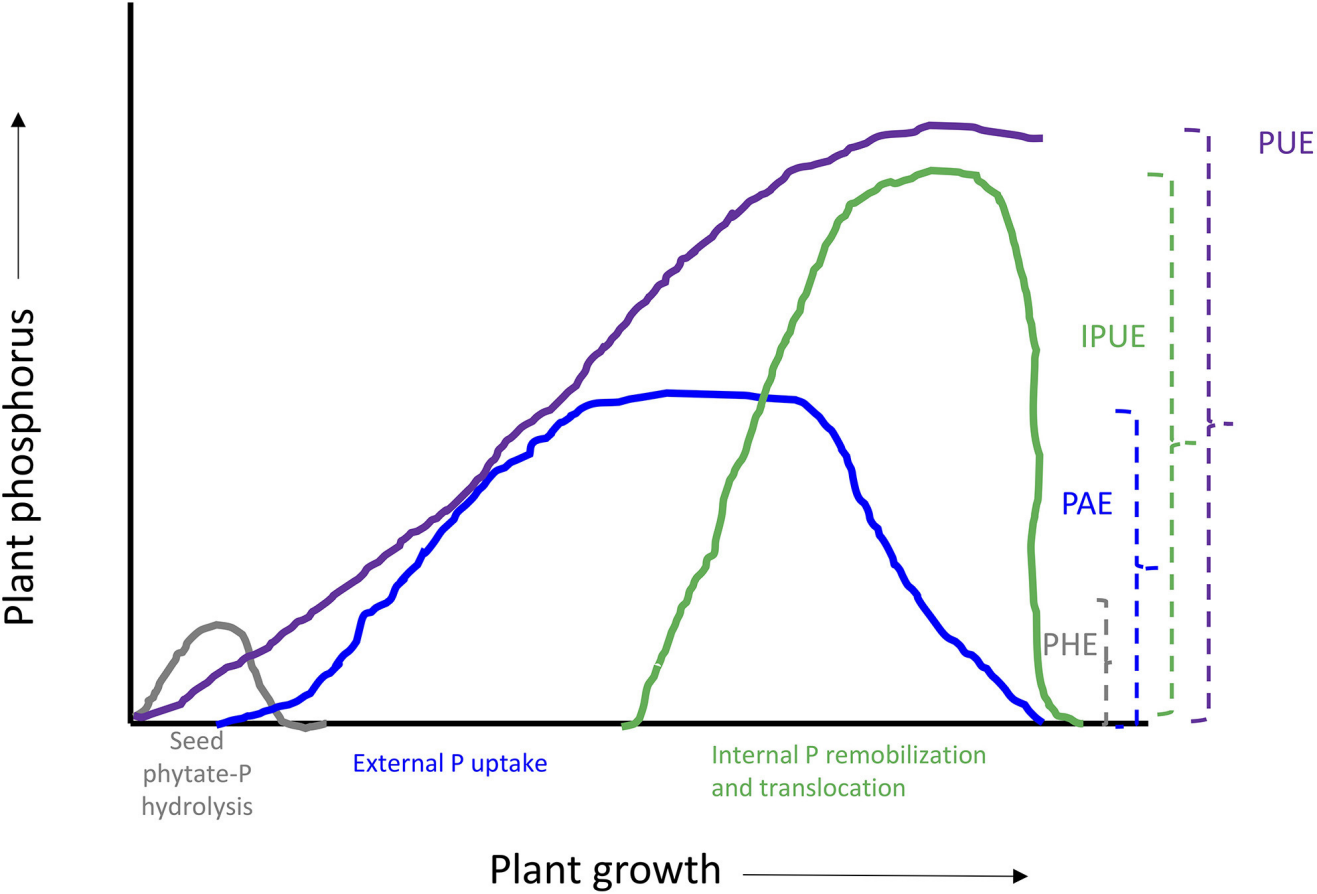
Schematic representation of plant phosphorus use efficiency and different components. PUE, phosphorus use efficiency; IPUE, internal phosphorus utilization efficiency; PAE, phosphorus acquisition efficiency; PHE, seed phytate-P hydrolysis efficiency.

Mineral nutrition efficiency concepts on crop plants have been defined based on how plants acquire essential mineral nutrients with the help of an underground extensive root system, mineral transport within a plant body, their translocation, storage, and remobilization, and re-translocation within plant organs to produce a desirable harvest (Ciarelli et al., 1998). Nutrient efficiency starts with the onset of ontogeny, which is marked with successful hydrolysis and remobilization of stored seed reserves (Figure 1), an important step for early seedling establishment and crucial for early plant development (White and Veneklaas, 2012). Therefore, the concept of nutrient use efficiency might be a combination of successful remobilization of heterotrophic stored seed nutrient reserves, autotrophic nutrient acquisition by roots or accumulation through shoots, and their internal translocation or re-translocation within actively growing plant organs (Figures 2, 3).

Many studies have been conducted to define different methods and means regarding plant nutrient use efficiencies; the most common is the ability of plants to convert nutrient inputs into biomass or minimize nutrient wastage in an agroecosystem (Batten, 1992; Lynch, 1998). The PUE of plants could be divided into PAE and IPUE; early seed P remobilization and seedling P nutrition are also an integral part of plant harvestable P (White and Veneklaas, 2012), particularly in soils with low inherent P status and seed P effluxes during germination (Nadeem et al., 2012). Therefore, higher PUE of plants could be achieved through improved seed with P reserves for better seedling establishment in P-deficient soils, P acquisition by root systems, and internal P utilization during vegetative and reproductive growth, or their combination (Shenoy and Kalagudi, 2005; Parentoni and Souza Júnior, 2008; Nadeem et al., 2011). The contribution from seed P from hydrolysis of phytate-P, PAE, or IPUE to PUE varies with crop species, phytate-P content in seeds, soil, and environmental factors. Modern agricultural practices include selecting crop genotypes with higher-quality seed with better nutrient stock (biomass) to support the early seedling establishment and adapt to scavenge soil P by improved root architecture and morphology that facilitate PAE (Vance et al., 2003). Some genotypes express higher P uptake capacity (Ciarelli et al., 1998; Corrales et al., 2007; Parentoni and Souza Júnior, 2008); however, low remobilization and re-translocation of P by plants is a challenge to harvest-acquired P (Figure 2). Therefore, it becomes important to explore better strategies that could improve seed P remobilization and translocation to newly growing seedlings while minimizing P effluxes (Nadeem et al., 2011), better PAE through the root system, and higher IPUE during the growing season. Here, we discuss different strategies of three important plant PUE contributors and possible improvements in podzolic soils in boreal climate.

### Seed Phytate-P Hydrolysis, Remobilization, and Use Efficiency

Phosphorus is highly mobile in plants and exists as free inorganic orthophosphate or organic phosphate ester; however, in seeds, P is loaded and stored in the form of phytate-P during crop maturity (Raboy, 1997). Simultaneously, P and cellular P are other forms of P in seeds and are generally referred to as available P (Raboy, 1997). As the primary storage form of P in seeds (Figure 1), phytate-P represents 50–90% of total P in mature seeds and accounts for 0.5–5% of seed dry weight (Greiner and Egli, 2003; Nadeem et al., 2011). In maize grains, phytate-P is mainly located in the scutellum compared to the endosperm, which is rich in carbon (C) and nitrogen (N) (Nadeem et al., 2011). It has been reported that mature seeds or grains contain enough phytate-P to provide P to sustain root and shoot growth for several weeks in developing maize seedlings (Nadeem et al., 2011), and this could be a potential point to enhance seed P content for prolonged seed P contribution in developing seedlings on P-deficient podzol soils for improving PUE (Figure 1). Phytate-P hydrolysis is also considered to provide other bound mineral nutrients to developing seedlings during the seedling establishment stage (Lott et al., 1995). We demonstrated earlier that phytate-P hydrolysis in seeds is not a limiting factor for P availability in germinating seeds for seedling P nutrition (Nadeem et al., 2012), and seedlings start to uptake significant external P soon after the emergence of radicle contributing to PUE (Nadeem et al., 2011). Boreal agroecosystems are characterized by a cool, short growing season particularly during early growth and crop maturity. Cool growing conditions during germination and early growth conditions affect seedling establishment because of low crop heat unit accumulation (Kwabiah et al., 2003), affecting biomass allocation to the shoot, which might further enhance P effluxes during early growth stages in podzolic soils. Such P effluxes depend on seed P reserves, variations among growing seedlings (Nadeem et al., 2012), and seedling growth-limiting conditions (Ali et al., 2019a), which could further reduce PUE in early crop growth stages in podzolic soils in boreal agroecosystems. Thus, initial heterotrophic P nutrition supported by seed P reserves is essential for early seedling establishment when external P uptake is restricted because of unavailability in P-deficient soils (White and Veneklaas, 2012), particularly in podzolic soils (Kedir et al., 2021). Therefore, enhancing phytate-P concentrations in seeds could have beneficial effects on early seedling success in podzolic soils; however, such an approach might increase P effluxes (Nadeem et al., 2012) in germinating seeds and growing seedlings because of low C assimilation under cool climatic conditions.

### Phosphorus Acquisition Efficiency

Following successful seed germination, growing seedlings depend on autotrophic P in soil acquired by developing root systems (Hinsinger, 2001). Seedlings absorb only small quantities of external P during the first few weeks of early growth (Nadeem et al., 2011); however, this early P nutrition is extremely important to enhance PUE and final crop harvest. Therefore, after exhaustion of heterotrophic seed P reserves, PUE could be explained by considering the role of various root physiological traits and biochemical processes in the rhizosphere zone and P availabilities in podzol soils (Figure 3). Plants have evolved several mechanisms to scavenge unevenly distributed P in the soil profile and uptake against a concentration gradient (Bieleski, 1973; Hinsinger, 2001; Ho et al., 2005). The main mechanisms associated with higher PAE include changes in root morphology, architecture, and functions of mycorrhizae hyphae that help plant roots to explore the rhizosphere zone, particularly in shallow natured podzol soils (Figure 3). The genetic ability of plants to associate with soil rhizosphere microorganisms, such as mycorrhizae, bacteria, and fungi (root symbiosis), and plants' inherent capacity to sense and secrete different organic compounds (root exudates) in the rhizosphere, such as phosphatases and organic acids, to release P from organic or inorganic soil sources (Raghothama, 1999). Here, we discuss the inherent ability of plants to scavenge P by plant root systems and different potential strategies to enhance PAE.

#### Root Morphology

Roots are the primary source to acquire the nutrients essential for growth and development in both monocot and dicot plants (Gregory, 2006). Efficient three-dimensional configuration of the root system is vital for improved nutrient uptake (Mollier et al., 2008; Li et al., 2016). Root growth and its developmental response are highly plastic and influenced by many factors such as soil P status, water availability, oxygen concentration, soil density, and pH (Péret et al., 2011, 2014). Growing plants respond to P deprivation in the root rhizosphere in several ways including allocating more C stocks to roots rather than shoots, thereby increasing root to shoot ratio, changes in lateral root formation (Mollier and Pellerin, 1999), longer roots, increased root hair density and length (Lynch and Brown, 2001), enhanced exudation of organic acids (Carvalhais et al., 2011), active microbial communities, higher acid phosphatase activities (Ali et al., 2019b), P transporters in roots (Shin et al., 2004; Kanno et al., 2016), higher association with mycorrhizae (Smith et al., 2011), formation of aerenchyma (Pujol and Wissuwa, 2018), as well as the growth angle of basal roots (Miguel et al., 2013). Such characteristics could enable roots to explore the rhizosphere in shallow podzolic soils to acquire the required plant P for optimum growth and development. However, not all crops have the ability to equally explore the rhizosphere P (Lynch and Brown, 2001; Williamson et al., 2001). Therefore, improved root morphological traits can play an important role in enhancing P uptake in shallow podzolic soils rich in Al^3+^ and Fe^2+^ minerals in boreal agroecosystems. Here, we briefly discuss root architecture and morphological traits that contribute to higher PAE in podzolic soils in boreal agroecosystems.

#### Root Architecture

The spatial configuration of the root system in the rhizosphere is often referred to as root architecture. It relates root distribution patterns and root topology in response to soil physiochemical properties including any nutrient deficiency (Lynch, 1995; Lynch and Brown, 2001). Therefore, root architecture determines the extent of root exploration capacities throughout the soil (Péret et al., 2014). Such plant strategies in the root architecture could occur at low C cost resulting in enhanced PAE (Wang et al., 2004a; Yan et al., 2004). Phosphorus is a major nutrient that is influenced by root architecture and development (Williamson et al., 2001). Modifying root architecture is an important strategy to improve PUE under P-deficient podzol soil conditions, allowing for improved plant performance (Li et al., 2016). The shallowness of podzolic soils might affect P uptake since upper soil layers are often rich in P; therefore, plant adaptation in root architecture and density might (van de Wiel et al., 2016) help to enhance PAE in podzolic soils. Additionally, root architecture adaptations including horizontal growth of basal-root, shallower growth angles of axil roots, increased adventitious roots, enhanced lateral root formation, and increased root hair density result in enhanced axial root lengths (Lynch and Brown, 2001; Vance et al., 2003; Lynch, 2007; Richardson et al., 2011; van de Wiel et al., 2016). Campos et al. (2018) demonstrated that these adaptations improve PAE in the topsoil under low-mobility P conditions that could be employed in podzolic soils. Lynch (1995) demonstrated that PAE is positively associated with basal root branching angles and the number of lateral branching roots per unit root area in common bean, especially under low P conditions. The shallow growth angle of axial roots increases PAE by increasing topsoil foraging in most soils (Lynch, 2011) and possibly in podzolic soils with shallow nature. Scavenging capacity in the upper soil layer increases with changes in growth angle and lateral root density (Ao et al., 2010).

Zhao et al. (2004) also reported a critical role of root shallowness in scavenging P in growing soybean for higher PAE, and, therefore, considered that podzolic soils might provide higher adventitious roots and lateral root growth to enhance PAE. However, in the study of Mori et al. (2016), shallow root angle was not beneficial, a genotype of rice with a steep root angle (37°) had a similar ratio of total root biomass (78%) compared with a shallow-rooted genotype (17°) (82%) in that soil top layer. This is probably due to the shallowness being related with root mass in 5 cm topsoil, but not with root biomass above 20–25 cm. Another important aspect is localized P supplies that can further enhance grass root proliferation to maximize PAE. Selecting crop genotypes with better root architectural traits would not only increase exploration of greater rhizosphere area but could also provide opportunities to uptake unevenly distributed P in podzol soils. Therefore, minor changes in root architecture could result in greater differences among different crop genotypes for PAE (Wissuwa, 2003), which could provide a better solution to scavenge P in podzolic soils. Mori et al. (2016) confirmed that a large root system is highly correlated with reduced P availability in soils. They also reported that root size and root efficiency represent two distinct tolerance mechanisms that increase plant performance and P uptake under P deficiency conditions.

#### Root Hair

Root hair formation from trichoblast, a tubular-shaped root cell (Gilroy and Jones, 2000; Singh et al., 2008), is a root architectural characteristic and plays a critical role in acquiring essential plant nutrients (Gilroy and Jones, 2000; Wang et al., 2016). Root hair is a low-cost way to explore a greater rhizosphere area and is a major contributors to nutrient uptake in plants (Wang et al., 2016). Parker et al. (2000) reported that about 77% of root surface areas are composed of root hair in Arabidopsis. Alteration in root tip growth and root length extension provides more surface areas to uptake nutrients from soils. Root hair formation and growth are associated with less mobile nutrients such as P, and an earlier study demonstrated that increased lateral root development and root hair intensity are related to higher PAE (Gilroy and Jones, 2000; Jungk, 2001; Wang et al., 2016). Ma et al. (2001) reported that P-deprived growth conditions improved trichoblast number, which likely increased root hair formation. Richardson et al. (2011) suggested allocation of carbon for root hair formation as a relatively minor plant metabolic cost to enhance P efficiency. Researchers demonstrated that the number of root hair and length are correlated with P concentration. For instance, the density of root hair in P-deficient soils is five-fold higher than in P-enriched soils (Bates and Lynch, 2000; Ma et al., 2001). Higher root hair density and total root length in soybean in basal roots than in taproots indicated highly positive correlations with root hair density and P concentration in soybean (Wang et al., 2004a). Similarly, common beans also expressed higher root hair density and longer root hair length in P-efficient genotypes (Yan et al., 2004). Moreover, Miguel et al. (2015) demonstrated phene synergism between root hair length and basal root growth angle for P uptake.

Sugars, auxin, and ethylene have been well-documented for their roles in modulating root hair formation during P deficiency (Niu et al., 2013; Song and Liu, 2015). Response to P deficiency in shoots causes signals in roots to enhance root hair elongation and abundance (Jungk, 2001). Parry (2018) reported that auxin homeostasis is crucial for root hair elongation under low external P availability conditions. Auxin in the lateral root cap and epidermal cells transported by AUX1 is satisfied for low-P induced root hair elongation. Giri et al. (2018) demonstrated that *OsAUX1*, the ortholog of *Arabidopsis* AUX1 sequence, functions in rice to mobilize auxin from root apex to differentiation zone to promote root hair elongation and improve PAE under low-P conditions. Bhosale et al. (2018) showed that the root hair elongation process was disrupted in auxin synthesis (*TAA1*) and transport (*AUX1*) mutants under P starvation conditions. In addition, auxin response transcriptional factors such as ARF19, RSL12, and RSL14 are also induced in response to low P (Bhosale et al., 2018). Similarly, about 40 genes have been identified in Arabidopsis that affect the initiation and development of root hair based on P availability in a growing medium (Grierson et al., 2001). Plant genotypes exhibiting higher changes in auxin for better root hair formation to explore more root rhizosphere can enhance PUE in acidic soils. Therefore, selecting crop genotypes with a better ability to enhance root hair formation could provide a better way to enhance P uptake from podzolic soils in boreal agroecosystems.

#### Proteoid Roots

Proteoid or cluster root formation is considered a major adaptation in plant species to enhance P uptake (Shane et al., 2003; Abdolzadeh et al., 2010; Cheng et al., 2011; Niu et al., 2013), particularly when P is present in sorbed or insoluble forms in soils (Lambers et al., 2006). Marschner et al. (1989) explained for the first time that extractability of nutrients is increased by several folds in the rhizosphere of proteoid roots. On the contrary, plants in P-rich soils do not invest energy for cluster root formation (Shane et al., 2003, 2006; Abdolzadeh et al., 2010). P deficiency in soils causes signal transduction from shoots to roots for cluster root formation and a variety of genes (*Suc synthase, triose-P isomerase, NAD-dependent glyceraldehyde 3-P dehydrogenase, enolase*, and *phospho*enol*pyruvate carboxylase)* are involved in such process. This process causes changes in primary and secondary metabolism, and P scavenging and remobilization in *Lupinus albus* L. (Uhde-Stone et al., 2003). Cluster root formation is important in *Leguminosae* and *Proteaceae* families, especially in P-deficient soils, and common in plant species that lack mycorrhizal association. The morphological and physiological characteristics of cluster roots enhance P uptake by releasing protons and carboxylates in the rhizosphere (Cheng et al., 2011; Lambers et al., 2012a). As such, cluster roots geared to enhance root surface area, exude significant amounts of carboxylates as exudative burst, generate localized concentrations, forage insoluble forms of soil P, and enhance P uptake (Lambers et al., 2006). Exudation of organic acids, such as malate and citrate, by cluster roots (Marschner et al., 1986; Massonneau et al., 2001) in podzolic soils could help in P release from bounded to plant-available form. Therefore, considering crop species with higher abilities and investment in cluster root formation could result in higher PAE and PUE in P-deficient soils compared to species lacking cluster root formation abilities. Such plant root characteristics would be very useful in agricultural production systems on acidic soil to enhance crop growth and biomass production with improved PUE.

#### Root Exudates

Plant roots exude a variety of organic compounds in the rhizosphere under normal growth conditions, which leads to a wide array of processes such as plant-microbe signaling, nutrient availability, and plant nutrient uptake (Harrison, 1997; Ali et al., 2019b). Root exudates include organic acids, phenolics, amino acids, sugars, proteins, and polysaccharides (Ryan et al., 2001; Marschner, 2011; Nadeem et al., 2016); however, these exudates vary in terms of quantity and quality under abiotic or biotic stress (Ryan et al., 2001; Neumann and Martinoia, 2002; Marschner, 2011). For instance, in P-deficient environments, growing plant roots show enhanced synthesis and exudation of several organic acids such as citric acid, malate, malic acid, cis-aconitic acid, and oxalic acid (Dinkelaker et al., 1989; Delhaize et al., 1993; Delhaize and Ryan, 1995; Larsen et al., 1998; Neumann et al., 2000), which induce changes in rhizosphere chemistry, soil microbial populations, and plant growth (Ali et al., 2019b). Ryan et al. (2001) explained that root exudates enhance phosphatase enzymes and P acquisition in growing plants. The release of organic acids in the rhizosphere displaces or mobilizes P from complex metal cations of soil colloids that compete for adsorbed P, thereby releasing P into the soil solution for plant uptake (Hinsinger, 2001; Ryan et al., 2001). Similarly, organic acids in root exudates destabilize organic matters and promote the cycling of organic P and P into soil solutions (Menezes-Blackburn et al., 2016). Vance et al. (2003) reported 20-to 40-fold higher citrate and malate exudates by cluster root system in P-deficient soils. Malate, citrate, and oxalate exudates are enhanced by lupine roots under P-deficient growth conditions. Citrate is considered most effective in releasing P through chelation of Al^3+^ in acidic soils (Menezes-Blackburn et al., 2016). Ström et al. (2002) reported enhanced P uptake by exogenous application of oxalate and citrate into maize plant rhizosphere, and such exogenous application upregulated P diffusion coefficient by three times in a soil solution. Enhanced citrate exudation by transgenic tobacco plants increased P acquisition, leading to enhanced PAE (López-Bucio et al., 2000). Higher acid phosphatase activities and P uptake were also observed in manure-applied corn silage under field conditions on podzolic soils in a boreal agroecosystem (Ali et al., 2019b). Such enhanced uptake due to root exudates contributes to increased PUE in growing plants. Crop genotypes exhibiting higher abilities of root exudation to cope with lower soil P availability could provide a sustainable solution to forage P and enhance PAE on podzolic soils in boreal agroecosystems.

#### Root Symbiosis

Arbuscular mycorrhizal fungi (AMF) and plant root symbiotic association is ubiquitous and plays an important role to enhance PAE in growing plants on P-deficient soils (Haran et al., 2000; George and Richardson, 2008; Adesemoye and Kloepper, 2009; Smith and Read, 2010). AMF colonize plant roots to obtain energy and, in exchange, release phosphate from arbuscular hyphae for plant uptake. Under P deficient conditions in the rhizosphere, AMF colonization promotes root growth by increasing the length and number of lateral roots and root biomass (Qiang-Sheng et al., 2011; Wu et al., 2011a; Chun-Yan et al., 2014). Fungi enter the inner cortex of roots and form highly branched hyphae enveloped by the root cell plasma membrane, thus forming an efficient signaling and nutrient exchange interface. Drew et al. (2003) reported that fungi also develop an extensive network of hyphae outside roots, which can easily penetrate into soil micropores. Li et al. (1991) reported that these effects are mainly due to the ability of AMF hyphae to scavenge inorganic P well beyond the limits of the rhizosphere depletion zone depending on plant genotype, P nutrition, and growth stage. Ryan and Graham (2002), however, documented that such roles of AMF are not always beneficial to growing plants because of greater carbon cost where they act as a parasite. Researchers demonstrated that 70–90% of plant species including ferns, bryophytes, flowering plants, and most agricultural crop plants can develop interdependent connections with AMF (Zhu et al., 2010; Ahanger et al., 2014; Begum et al., 2019; Diagne et al., 2020). Hidden mechanisms are not fully explored; however, AMF contributions to growing plants in terms of plant P nutrition and plant health and fitness are well-documented (Smith and Read, 2010; Plassard et al., 2019). Battini et al. (2016) demonstrated that a culture-dependent approach allows for the isolation in a pure culture of 374 bacterial strains that are associated with plant growth-promoting activities such as phytate-P mineralization and solubilization of P. These positive effects of AMF association with plants are essential for upcoming challenges in terms of enhanced PAE and PUE in podzolic soils for sustainable crop production.

Beneficial microorganisms also play a vital role in the biogeochemical cycling of organic and inorganic P in the rhizosphere (Harvey et al., 2009; Khan et al., 2010; Richardson and Simpson, 2011). Researchers demonstrated that plant root inoculation with plant growth-promoting bacteria (PGPB) enhanced soil organic P, e.g., inositol phosphates (Richardson et al., 2011; Pereira et al., 2020). These bacteria with extracellular phytase activity and their interactions in the rhizosphere increase plant access to P from inositol phosphates (Unno et al., 2005; Richardson and Simpson, 2011) and improve plant PUE. Microbial inoculants in the rhizosphere have been known as systems of integrated nutrient management, with a specific interest in their ability to enhance the availability of P for crops (Richardson et al., 2011). Bacteria (most commonly Azospirillum, Bacillus, Pseudomonas, and Actinomycetes) have been able to release orthophosphate from organic and inorganic substrates by liberating protons, organic anions (e.g., citrate, oxalate, and gluconate), phosphatases, and cation-chelating compounds (El-Tarabily et al., 2008; Taurian et al., 2010). However, performing microbial inoculation on P-solubilizing fungi, especially species of Penicillium (*Penicillium radicum and Penicillium bilaiae*), has been the main effort to increase P availability (Wakelin et al., 2007; Richardson and Simpson, 2011). Generally, the major mechanism of PGPB may be related to the production of phytohormones such as auxins, salicylic acid, cytokinins, and gibberellins, and to P solubilization that enhances density and length of roots and increases a plant's ability to capture available nutrients (Pereira et al., 2020).

#### Phosphate Transporters

The movement of P in soils is mainly dependent on diffusion, so plants themselves contribute to the spatial heterogeneity of P by depleting it from the rhizosphere (Miguel et al., 2013). Poirier et al. (1991) documented the concentration of P in the xylem membrane to be as high as 10 mM, so an active P transport mechanism is required to uptake P by growing roots and within the plant body. In recent years, two different groups of P transporters have been cloned for growing plants with either low-affinity or high-affinity P transporters. Low-affinity P transporters operate in the chloroplast membrane (Versaw and Harrison, 2002), whereas the high-affinity ones (Km ranging from 50 to 300 μM) are expressed in root epidermal cells during growth under low P conditions (Harrison et al., 2002; Raghothama and Karthikeyan, 2005). This active transport system comprised of a high-affinity and energy-rich transport mechanism is pivotal (Smith et al., 2003) for P uptake from a soil system (Figure 2). Mimura (1995) demonstrated the existence of an active transport system as well as channel activity, which enables the translocation of P across the tonoplast membrane during uptake in the vacuole. So far, P-H^+^ co-transporters located in the plasma membrane belong to the *PHT1* gene family, and most of them are expressed in root cells (Table 2). Raghothama and Karthikeyan (2005), and Smith et al. (2003) explained that under P-deprived conditions, *PHT1* genes are significantly induced to enhance the ability to grow roots to uptake P from soil as well as to re-translocate it within growing plant compartments (Table 2). In contrast, repression of high-affinity *PHT1* genes could alleviate P toxicity under high P-concentration soil conditions (Dissanayaka et al., 2020). The family of *PHT1* transporters has been identified in several plant species (Table 2), and most of its members exhibit strong expression in roots and possess a range of affinities for P uptake (Nussaume et al., 2011). For instance, there are 9 *PHT1* genes identified in Arabidopsis where *AtPHT1;1* and *AtPHT1;4* is highly expressed at the root-soil interface, including root hair, root cap, and epidermis cells under P deprivation conditions (Mudge et al., 2002). All these major genes are responsible for P uptake by roots from P-enriched or deficient rhizospheres (Misson et al., 2004; Shin et al., 2004; Catarecha et al., 2007). Kanno et al. (2016) employed a real-time ^33^P microimaging technique and showed that root cap accounts for 20% of total *Arabidopsis* seedling P uptake. Moreover, compared to wild-type and complemented lines, the expression of P-induced genes is higher in impaired P, and they function together in the endoplasmic reticulum of root tips that mediate root meristems during P deprivation (Ticconi et al., 2009). Recently, Balzergue et al. (2017) identified a new root growth inhibition signaling pathway, STOP1-ALMT1, under low-P stress in Arabidopsis. The authors proposed that P limitations enhance STOP1 abundance to activate ALMT1 expression that further promotes malate exudation by root apex internal cell layers that rapidly inhibits root cell elongation (Balzergue et al., 2017).

**Table 2:** Inorganic phosphorus transporter genes in different plant species.

**Plant species**	**Botanical name**	**Identified *PHT* genes**	**Location**	**References**
Arabidopsis	*Arabidopsis thaliana* L.	*AtPT1, AtPT2, AtPht1;4, AtPHT1.8, AtPHT1.9*	Roots and roots to shoot	Muchhal et al., 1996; Mudge et al., 2002; Smith and Read, 2010; Lapis-Gaza et al., 2014
Barley	*Hordeum vulgare* L.	*HvPHT1;1, HvPHT1;2, HvPht1;3, HvPht 1;4, HvPht 1;5, HvPht1:6, HvPht1;7, HvPht1;8, HvPht1;11*	Epidermal and cortex cells, vascular root tissues, shoot and older leaves, roots and AMF associations	Rae et al., 2003; Smith et al., 2003; Schünmann et al., 2004; Glassop et al., 2005; Preuss et al., 2010
Stiff brome grass	*Brachypodiumdistachyon L*.	*BdPT1, BdPT3, BdPT4, BdPT7, BdPT8, BdPT9, BdPY10, BdPT11, BdPT12, BdPT13*	Roots and shoots	Hong et al., 2012
Eggplant	*Solanum melongena* L.	*SmPT1, SmPT2, SmPht2;1, SmPT3, SmPT4, SmPT5*	Leaves, roots and AMF association	Chen et al., 2007
Foxtail millet	*Setariaitalica L*.	*SiPHT1;8, SiPHT1;9*	Roots with AM fungi association	Ceasar et al., 2014
Maize	*Zea mays* L.	*ZmPHT1;1, ZmPHT1;2, ZmPHT1;3, ZmPHT1;4, ZmPHT1;6*	Roots, stem, anther, silk, seed, cob, young, old leaves and AMF association	Glassop et al., 2005; Nagy et al., 2006; Liu et al., 2016a
Medicago	*Medicago truncatula* L.	*MtPT1, MtPT2, MtPT3, MtPT4, MtPT5*	Roots epidermis, cortex, vascular tissues and periarbuscular tissues	Liu et al., 1998, 2008; Chiou et al., 2001; Harrison et al., 2002
Chinese milk vetch	*Astragalus sinicus L*.	*AsPT1, AsPT2, AsPT3, AsPT4, AsPT5*	Arbuscule-containing cell of the cortex, root epidermis, cortex and stele cells	Xie et al., 2013; Fan et al., 2020
Pepper	*Capsicum frutescens L*.	*CfPT1, CfPT2, CfPht2;1, CfPT3, CfPT4, CfPT5*	Roots, leaves and AMF association	Chen et al., 2007
Potato	*Solanum tuberosum* L.	*StPT1, StPT2, StPT3, StPT4, StPT5*	Leaves, tubers, roots, floral organs, periarbuscular membraneand AMF association	Leggewie et al., 1997; Rausch et al., 2001; Nagy et al., 2005
Rice	*Oryza sativa* L.	*OsPht1;1, OsPHO1;1, OsPHO1;2, OsPht1;2, OsPHT1.4, OsPht1.6, OsPHT1.8, OsPht1;11*	Stele, primary and later roots, root tips, exodermis root layers, AMF association, leaves, ligules, stamens, caryopses, xylem and phloem	Paszkowski et al., 2002; Seo et al., 2008; Ai et al., 2009; Jia et al., 2011; Sun et al., 2012; Ye et al., 2015; Che et al., 2020
Soybean	*Glycine max L*.	*GmPT1, GmPHT1;14, GmPT2*	Roots and shoots	Wu et al., 2011b; Fan et al., 2013
Tomato	*Solanum lycopersicum*L.	*LePT1, LePT2, LePT3, LePT4, LePT5*	Epidermis and central cylinder cells of roots, palisade parenchyma, phloem cells of leaves and arbuscules-containing cell of cortex and leaves	Daram et al., 1998; Liu et al., 1998; Nagy et al., 2005; Gómez-Ariza et al., 2009
Tobacco	*Nicotiana tabacum* L.	*NtPT1, NtPT2, NtPht2;1, NtPT3, NtPT4, NtPT5*	Roots, leaves, stems and AMF association	Kai et al., 2002; Chen et al., 2007; Tan et al., 2012
Wheat	*Triticum aestivum* L.	*TaPHT1.1, TaPHT1.2, TaPHT1.3, TaPHT1.4, TaPHT1.4-5B, TaPHT1.5 TaPHT1.6, TaPHT1.6-5A, TaPHT1.7, TaPHT1.8, TaPHT1.8-6A, TaPHT1.9, TaPHT1.10, TaPHT1.10-4A, TaPHT1.10-U, TaPHT1.11-4A, TaPHT1.11-4B, TaPHT1.11-4D, TaPHT1.12-7A, TaPHT2;1, TaPHT3;1*	Roots. Leaves, stem and AMF association	Davies et al., 2002; Glassop et al., 2005; Sisaphaithong et al., 2012; Huang et al., 2013; Teng et al., 2013, 2017; Aziz et al., 2014

Transcription factor STOP1 (*sensitive to proton toxicity 1*) induces *ALMT1 (aluminum-activated malate transporter-1)* expression followed by cell elongation rapid inhibition by peroxide-dependent transportation lines (*phf1-2*) in a P-rich medium (Kanno et al., 2016). A previous study has identified a QTL that encodes ferroxidase, *LPR1* (*low phosphate root 1*), which controls root growth under P starvation conditions in *Arabidopsis thaliana* (Reymond et al., 2006). β-glucuronidase (GUS) staining and qRT-PCR analysis showed that *LPR1* is mainly expressed in root tips, and that loss of function of *LPR1* and its close paralog *LPR2* lines did not arrest primary root growth in a low-P medium (Svistoonoff et al., 2007). *LPR1* could counteract *PDR2* phosphate deficiency and cell wall stiffening in the stem cell niche and root apical meristem, suggesting that Fe accumulation mediated by malate is a crucial checkpoint for root development in response to P deficiency (Balzergue et al., 2017; Mora-Macías et al., 2017). Defects in P uptake in a growth medium under low P conditions have been noticed in *AtPHT1;1* and *AtPHT1;4* mutants (Misson et al., 2004; Shin et al., 2004). In rice, 10 out of 13 *PHT1* genes were expressed in roots (Table 2), whereas *OsPHT1;11* expression was exclusively induced by mycorrhiza symbiosis (Paszkowski et al., 2002). Ai et al. (2009) reported a significant increase in OsPHT1;2 and OsPHT1;6 in roots of rice under P deprived growth conditions. Similarly, 11 *PHT1* genes have been reported in barley roots under P deprivation conditions, as shown in Table 2 (Preuss et al., 2011; Sisaphaithong et al., 2012). In addition to *OsPHT1* genes, PHOSPHORUS-STARVATION TOLERANCE1 (OsPSTOL1) has been found to be up-regulated in root tissues and shown to increase P uptake under P-deficient conditions because of enhancement of early root growth and development (Gamuyao et al., 2012). The homologs of *OsPSTOL1* in sorghum (SbPSTOL1) also alter root morphology and root system architecture, and significantly improve grain yield under low P conditions (Hufnagel et al., 2014). PSTOL1 proteins possess structural features of wall-associated kinase (WAK) proteins such as Ser/Thr kinase, transmembrane, and extracellular domains important for their functionality in the cell wall (He et al., 1999). Analysis of single-copy *Ds* transposon mutant repository of barley (Singh et al., 2006, 2012; Bregitzer et al., 2019) revealed that WAK proteins are implicated in the root growth of barley (Kaur et al., 2013; Tripathi et al., 2021), and that they are potentially new gene candidates for P starvation tolerance.

Post-translational regulations, such as ubiquitination and phosphorylation, are also involved in controlling *PHT1s* (Table 2). In Arabidopsis, the E2 ubiquitin conjugate UBC24/PHOSPHATE 2 (*PHO2*), regulated by miRNA399, could interact with *PHT1s* in the endoplasmic reticulum and mediate their ubiquitination, thereby regulating *PHT1s* activities negatively toward the plasma membrane under P-sufficient conditions (Bari et al., 2006; Chiou et al., 2006; Huang et al., 2013). Nitrogen limitation adaption (NLA) is a ring-type ubiquitin E3 ligase, and transcripts of NLA are targeted by miRNA827 and required for the degradation of *PHT1;1* and *PHT1;4 via* ubiquitination (Peng et al., 2007; Pant et al., 2009). Loss of function of the NLA mutant increases the protein level of *PHT1s* in the plasma membrane (Lin et al., 2013). Previous studies have shown that *PHT1* proteins require a phosphate transporter traffic facilitator (*PHF1*) to exit the endoplasmic reticulum (González et al., 2005; Bayle et al., 2011), but phosphorylation of *PHT1s* will inhibit their trafficking from the endoplasmic reticulum to the plasma membrane (Dissanayaka et al., 2020). Chen et al. (2015) indicated that Casein kinase-2 (CK2) could phosphorylate both low-affinity transporter *OsPT2* and high-affinity *PHT1* member *OsPT8* during P-sufficient conditions in rice, thereby inhibiting their interaction with *OsPHF1*. In contrast, the OsCK2β3 subunit is degraded during P-deficient conditions, which increases P uptake (Chen et al., 2015). Moreover, OsCk2 could also phosphorylate *OsPHF1* and *OsPHO2* to modulate P homeostasis in rice (Wang et al., 2020; Yang et al., 2020). Plant species with an active transport system with higher expressions of transporter are required to enhance PAE and PUE in P-deficient soil and represent promising targets for engineering high PUE crop genotypes.

## Internal Phosphorus Utilization

Internal phosphorus utilization efficiency (IPUE) is defined as the biomass produced per unit of P that accumulated in tissues. Higher IPUE has been attributed to optimum P translocation to harvestable plant parts by P re-translocation through phosphatase activities, P transporters (Table 2), and related mechanisms to transfer P from shoots to grains in different crop species (Parentoni and Souza Júnior, 2008; Rose and Wissuwa, 2012; Li et al., 2017; Adem et al., 2020). Plant P status largely determines IPUE through allocation and remobilization processes from different plant compartments. During the vegetative growth period, P uptake by the root system is continuous (Figure 3); however, remobilization and re-translocation of P from older/senescence tissues can become a very significant internal P source for new developing organs such as young leaves and grains (Figure 3). Therefore, during reproductive growth, remobilization and re-translocation from senescence vegetative tissues are typically the primary source of P for sink tissues as uptake by the root system starts declining and available soil P is depleted (Glassop et al., 2005; Rose and Wissuwa, 2012; El-Mazlouzi et al., 2020). Therefore, the major contributing traits in IPUE include P remobilization, P transporters, phosphatase activities, P release from vacuoles, and alternative biochemical pathways in different plant organs (Figures 2, 3).

### Phosphorus Remobilization

Efficient P remobilization and re-translocation within plant parts are attributed to higher IPUE in growing plants. Remobilization of P from senescent vegetative tissues is critically important to plant growth and development (Veneklaas et al., 2012), particularly to soils with low P availability. Phosphorus re-translocation is assisted by specific transporters, namely, the *PHO1* family (Table 2). Teng et al. (2017) reported that the Pht1 family of P transporters play an important role in the transport process across plant membranes (Table 2). Plants can remobilize more than 50% of P from senescent leaves (Aerts, 1996). However, similar to seed P contributions during early growth stages, potential P benefits from senescent vegetative parts are significantly smaller during the active growth period (Veneklaas et al., 2012). During this active growth period, external P uptake is the prime source for higher PUE. However, with the decline in growth rate during the later growing season, remobilization of P from older vegetative parts could sustain new growth and development and leaf function (Robinson et al., 2012). Remobilization of P reserves from older vegetative parts to newly growing parts is critically important in soils with low inherent P or low P inputs from organic or inorganic fertilizers. For instance, El-Mazlouzi et al. (2020) demonstrated that wheat plants grown under low P supply conditions translocate 72% of post-anthesis P uptake to grains compared to 56% share in plants grown with adequate P supply. Aerts (1996) demonstrated that growing plants tend to remobilize at least 50% of P from senescent vegetative parts. Akhtar et al. (2007) and Smith (2001) showed that re-translocation of P from metabolically inactive sites to actively growing plant compartments under low P conditions enhances PUE in *Brassica napus*. Similarly, the decreased growth rate under P deficiency conditions is always higher than the effects of senescence, making remobilization and re-translocation contributions significantly higher than uptake. Besides senescence, other plant species expressed the re-translocation of P in young and non-mature plant tissues showing remobilization and re-translocation recycling patterns (Akhtar et al., 2008; Nadeem et al., 2012). Moreover, a higher pre-translocation rate from shoots to roots impels tolerant *Brassica napus* cultivars to establish a better root system, thereby aiding plant growth under low P conditions and increasing PUE (Akhtar et al., 2008). Hence, to enhance PUE in growing crops on podzol soils in boreal ecosystems, it is important to understand and improve remobilization and re-translocation efficiency.

### Phosphate Transporters in Growing Plants

Phosphate-1 (*PHO1*) protein plays a critical role in the transfer of P from roots to shoots once P is assimilated by the roots (Wang et al., 2021). In Arabidopsis, the *PHO1* mutant has low P content in shoots and leaves due to a deficiency in loading P into xylem (Poirier et al., 1991). The Arabidopsis genome contains 11 members of the *PHO1* gene family, and they all have the same topology and harbor an SYG1/PHO81/XPR1 (SPX) domain in the N-terminal hydrophilic portion and an ERD1/XPR1/SYG1 (EXS) domain in the C-terminal hydrophilic portion, which shows different functions in plant P homeostasis (Wang et al., 2004b; Wege et al., 2016; Wild et al., 2016). There are three homologs in the rice genome, *OsPHO1;1, OsPHO1;2*, and *OsPHO1;3*, as shown in Table 2 (Secco et al., 2010). Two node-localized transporter members (*OsPHO1;1 and OsPHO1;2*) of the PHO1 family are involved in P allocation to seeds in rice (Che et al., 2020). *TOS17* insertion mutants of these two transporters showed lower grain yield and longer germination days because of impairment of P distribution to seeds (Che et al., 2020). Recently, Ma et al. (2021) identified the predominant role of OsPHO1;2 as a plasma membrane transporter in P reallocation during rice grain filling; OsPHO1;2 could export excess P from developing endosperm and unload P from pericarp into endosperm cells to maintain P homeostasis. The *Ospho1;2* mutant showed a poor grain-filling phenotype, and the activity of a starch synthesis enzyme, ADP-glucose pyrophosphorylase (AGPase), was also inhibited. Overexpression of *OsPHO1;2* enhances grain yield and PUE under P-deficient conditions in the field, which suggests that OsPHO1;2 is a target gene for enhancing grain yield for sustainable agriculture (Ma et al., 2021). In addition, PHO1 is a direct degradation target of PHO2 upon P resupply to P-starved plants (Bari et al., 2006; Liu et al., 2012). A recent research study indicated that an upstream open reading frame (uORF) of *PHO1* strongly inhibits translation of the main open reading frame (ORF; Reis et al., 2020). AUG-to-UUG point mutation of *PHO1* uORF enhances shoot P content and improves P deprivation response (Reis et al., 2020). Therefore, regulation of PHO1 expression *via* uORF editing shows a great potential for enhancing IPUE in different crops under P-deficient conditions (Table 2).

Intercellular re-distribution of P is regulated by four other phosphate transporter family members, *PHT2, PHT3, PHT4*, and *PHT5* (Table 2), which are localized to plastid membranes, mitochondrial membranes, Golgi compartments, and tonoplasts, respectively (Rausch and Bucher, 2002; López-Arredondo et al., 2014). The primary function of these transporters is the transportation of P to each organelle, and they are also coordinating cytosolic P homeostasis and various metabolic processes by controlling P exchange among different compartments. Low-affinity P transporters from the *PHT2* family are involved in the loading and unloading of P in vascular tissues, thereby affecting IPUE and overall PUE (Smith, 2001; Smith et al., 2003). In *Arabidopsis*, chloroplast envelope-localized *PHT2;1* was identified as the low-affinity chloroplast P transporter (Table 2). Mutant analysis showed that PHT2;1 could influence related gene expression and P allocation in plant leaves during P-starvation (Daram et al., 1999; Versaw and Harrison, 2002). PHT3 proteins are responsible for translocating P into the mitochondrial matrix, where it is used for adenosine triphosphate (ATP) synthesis (Młodzińska and Zboińska, 2016). Zhu et al. (2012) showed that overexpression of three *PHT3* genes (*AtPHT3;1, AtPHT3;2*, and *AtPHT3;3*) increased salt stress sensitivity by affecting ATP and gibberellin homeostasis in Arabidopsis. The *PHT4* family in the Arabidopsis genome has six members. Their locations vary and they are considered to have additional distinct functions (Guo et al., 2008; Miyaji et al., 2015). Karlsson et al. (2015) identified *PHT4;1* as a thylakoid transporter that functions in chloroplast phosphate compartmentation and ATP synthesis in the chloroplast stroma. *PHT4;6* is located in the Golgi apparatus, which plays a critical role in preventing plant dark-induced senescence caused by cytosolic P-starvation. The *PHT4;6* mutant showed low cytosolic P content and blockage of Golgi-related biosynthesis processes in *Arabidopsis* (Hassler et al., 2016), suggesting that *PHT4* proteins are involved in P transportation among cytosol, chloroplasts, non-photosynthesis plastids, and the Golgi apparatus (Guo et al., 2008). Therefore, crop species with an active transport system could perform better in the redistribution of P within plant compartments after uptake when plants are grown on podzolic soils in boreal agroecosystems.

### Phosphorus Re-translocation in Plant Parts

Plant leaves reserve P in various chemical forms, including nucleic acids, phospholipids, free P, and phosphorylated metabolites (Robinson et al., 2012). Intercellular and secreted ribonucleases (RNases) and acid phosphatases (APases) play an essential role in remobilizing P to growing plant parts from the degradation of organic P in senescent leaves (Dissanayaka et al., 2018). APases contribute to increased PUE in beans through P remobilization from old leaves (Akhtar et al., 2008). Also, APases produced by plants and microbes are involved in organic P degradation, which catalyzes the hydrolysis of easter-phosphate bonds and anhydrides to release P with an optimum acid pH at the 4.7–7 level (Raghothama and Karthikeyan, 2005; López-Arredondo et al., 2014). Purple acid phosphatases (PAPs) represent a specific group of APases, which hydrolyze P-ester to facilitate P use efficiency in plants (Ghahremani et al., 2019). Synthesis and secretion of PAPs have been identified in many crop species as a response to low P availability (Bozzo et al., 2006; Zhang et al., 2011; González-Muñoz et al., 2015; Li et al., 2017; Dissanayaka et al., 2018). The function of intercellular (vacuolar) PAPs is to recycle P from intercellular organic P pools. This process is accompanied by a decrease in cytoplasmic phosphate metabolites such as glycerol-3-phosphate, glucose-6-phosphate, and ATP under P-depleted conditions (Hurley et al., 2010; Tian and Liao, 2018). In the *Arabidopsis* genome, the *AtPAP* family is encoded by 29 genes and divided into three polygenetic groups according to amino acid sequences (Raghothama and Karthikeyan, 2005; Tran et al., 2010; Wang et al., 2014). Physiological functions of some *AtPAPs* genes were identified in transgenic plants. Wang et al. (2014) used all *AtPAP* mutants from *Arabidopsis* and showed that *AtPAP10* secreted APases, and that *AtPAP12* and *AtPAP26* isozymes were both intracellular and secreted APases. *AtPAP26* was reported to be a dual-targeted gene that played a vital role in maintaining P homeostasis in vacuolar P recycling and rhizosphere organic P utilization during P-deficient conditions (Veljanovski et al., 2006; Hurley et al., 2010). Loss of function of the *AtPAP26* mutant interferes in P remobilization metabolism during *Arabidopsis* leaf senescence status (Robinson et al., 2012). Also, OsPAP26 played dual functions (intercellular and extracellular) in rice; overexpressed *OsPAP26* plants increased P remobilization from senescent leaves to non- senescent leaves compared to *OsPAP26*-RNAi lines (Gao et al., 2017).

Nucleic acid pools make a great contribution for P scavenging and recycling in P-starved plants (Stigter and Plaxton, 2015; Dissanayaka et al., 2020), and ribosomal RNA (rRNA) is the largest organic P pool in mature leaves. During early leaf senescence and P deficiency, degrading RNA by some ribonucleases (Rnases) is considered a rescue system so P could be remobilized to young organs in higher plants (Tran et al., 2010; Robinson et al., 2012). Two of the S-like Rnase genes, *RNS1* and *RNS2*, belong to the T_2_/S superfamily and significantly induce mediating the response of low-P treatments and senescence. Higher content of anthocyanin was observed in antisense *RNS1* or *RNS2* transgenic *Arabidopsis* lines, which was the main symptom of P-deficiency in plants (Bariola et al., 1999). RNS1 was predicted as an extracellular protein, whereas RNS2 localized to the vacuole and endoplasmic reticulum (Poirier and Bucher, 2002; Hillwig et al., 2011). RNS2 can degrade RNA to 3'NMP; then, 3'-NMP is subsequently dephosphorylated by PAPs such as AtPAP26 or phosphorylases (Bassham and MacIntosh, 2017; Dissanayaka et al., 2018). rRNA degradation and turnover also depend on ribophagy (an autophagy-like process) that has been documented in yeast and plants (Kraft et al., 2008; Floyd et al., 2015; Huang et al., 2015). Higher levels of rRNA in vacuoles accumulated in *rns2 atg9* double mutant plants, which revealed that both S-like Rnase and autophagy were involved in an RNA turnover mechanism (Floyd et al., 2015). These findings suggest the importance of RNA as a P source for remobilization during P-starvation and late plant growth stages. However, the role of Rnase in RNA turnover and cellular P homeostasis in other crops needs to be further investigated.

Organelle DNAs from mitochondria and chloroplasts are known to be highly abundant and act as an internal reservoir of phosphate in plant cells (Oldenburg and Bendich, 2015; Takami et al., 2018). Degradation of organelle DNA by DPD1, a conserved and Mg^2+^-dependent exonuclease, was demonstrated to have a positive correlation with PUE during P-starvation and leaf senescence in Arabidopsis (Matsushima et al., 2011; Takami et al., 2018). T-DNA insertion *DPD1* mutants showed higher chloroplast and mitochondria DNA copy numbers than the wild-type plant and attenuated P derivation response (Takami et al., 2018). Therefore, the *DPD1* system showed a great engineering breeding potential for enhancing PUE in crop production.

As the second organic P source in plants, phospholipids account for 25% of the total organic P pool of a mature leaf (Veneklaas et al., 2012; Dissanayaka et al., 2018). Therefore, phospholipases also play an important role in P utilization by altering membrane lipid compositions (Shen et al., 2011). During P starvation, decreasing phospholipids and increasing non-phosphorus lipids such as galactolipid and sulfolipid, mostly digalactosyldiacylglycerol (DGDG), monogalactosyldiacylglycerol (MGDG), and sulfosulfoquinovosyldiacylglycerol (SQDG), could be the strategy for optimization of P utilization in plants (Härtel et al., 1998, 2000; Li et al., 2008). For instance, compared to *Arabidopsis*, six Proteaceae species from southwestern Australia in severely P-impoverished soil showed a higher percentage of galactolipids and sulfolipids during the vegetative stage (Lambers et al., 2012b). Expression of galactolipids and sulfolipid biosynthesis-related genes in younger leaves is generally up-regulated during P starvation in high-PUE rice genotypes (Adem et al., 2020). Diacylglycerol is considered an important intermediate in phospholipid degradation, which is the precursor for galactolipid biosynthesis. Phospholipases such as phospholipase C (PLC) and phospholipase D (PLD) have been suggested to be involved in the process of degradation. Low-P treatment and leaf senescence resulted in up-regulation of *PLC, PLD*, and phosphatide phosphatase (*PAH*) genes in plants (Hong and Lu, 2014; Jeong et al., 2017). Phosphatidic acid (PA) is generated by PLD and subsequently hydrolyzed by PAH to produce DAG and release P (Wang et al., 2006; Dissanayaka et al., 2018). Lipidomic analysis of *PLD*ζ*2* mutant *Arabidopsis* plants showed reduced capacity for galactolipid accumulation under P-limited stress (Cruz-Ramírez et al., 2006; Lin et al., 2020). Moreover, Cruz-Ramírez et al. (2006) found that both *PLD*ζ*1* and *PLD*ζ*2* were essential for lipid turnover by hydrolyzing phosphatidylcholine (PC) in *Arabidopsis* rosettes and roots for P deficiency acclimation. PLC can directly hydrolyze phospholipids to produce DAG. NPC4, a nonspecific PLC in *Arabidopsis*, was significantly upregulated during P deprivation. However, there was no apparent effect on phospholipid hydrolysis and DGDG biosynthesis in the *npc4* T-DNA insertion mutant (Nakamura et al., 2005). NPC5 was reported to be responsible for around 50% of DGDG biosynthesis in leaves by hydrolyzing plasma membrane phospholipids in response to P limitation (Gaude et al., 2008). Recently, Okazaki et al. (2013) found a novel class of plant lipid, glucuronosyldiacylglycerol (GlcADG), which accumulated under P-depleted conditions in *Arabidopsis* and rice. Moreover, in the biosynthesis of GlcADG that shared the same pathway of SQDG in chloroplast, the SQDG synthase mutant *sqd2* showed that neither SQDG nor GlcADG accumulated under P-starved conditions (Okazaki et al., 2013). Taken together, the research findings mentioned above revealed that these intercellular P pools may play a prominent role in internal P utilization when plants are facing P starvation.

### P Release From Vacuoles

Plants distribute P in different cell compartments selectively, such as for metabolic activities P is located in cytoplasts, whereas a vacuole is used for stored P pools (Theodorou and Plaxton, 1993). Therefore, vacuoles are known as major storage compartments under sufficient P supply conditions. Bieleski (1973) demonstrated that more than 70% of intercellular P was stored in vegetative plant vacuoles. The vacuole P in buffering cytoplasmic P maintains cellular homeostasis under short-term P starvation conditions (Yang et al., 2017). Therefore, translocation of P to and from a vacuole under P deficient conditions mainly maintains P homeostasis in growing plants. Luan and Lan (2019) demonstrated that vacuolar phosphate transporters (VPTs) play an essential role in buffering the level of cytoplasmic P to maintain cellular P homeostasis in response to changes in external P supply and metabolic demand. In yeast, *Saccharomyces cerevisiae, ScVTC1, ScVTC2, ScVTC3*, and *ScVTC4* are four vacuolar transmembrane proteins responsible for polyphosphate accumulation (Secco et al., 2012). In contrast, *ScPHO91* acts as an efflux transporter during P-starvation (Hürlimann et al., 2007). However, the transportation system from the tonoplast in plants is still unclear; tonoplast P transporters are not fully understood yet (Panigrahy et al., 2009). In a recent research study, the VPTs/phosphate transporter 5 (*PHT5*) family with an SYG1/PHO81/XPR1 (SPX) domain, as well as a major facilitator superfamily (MFS) domain, has been identified functioning as a vacuolar influx P transporter, which has three members in the *Arabidopsis SPX-MFS* family (Liu et al., 2015, 2016b). Loss of function of the *PHT5;1* mutant impairs P accumulation, and a decrease of 40% in P levels of the mutant line was observed compared to the wild type (Liu et al., 2016b). Similarly, the *OsSPX-MFS1* and *OsSPX-MFS3* homologs were also identified as a major tonoplast-localized influx P transporter in rice (Luan and Lan, 2019). Under P-deficient conditions, the influx of P from apoplast to the *PHT1* family and the efflux of P from vacuoles by vacuolar P efflux transporters (VPEs) facilitate cytosolic P homeostasis. In rice, Xu et al. (2019) identified two tonoplast-localized VPE proteins, which evolved from ancestral plasma membrane glycerol-3-phosphate transporter protein (GlpT), and named them *OsVPE1* and *OsVPE2*. Overexpression of either of these two genes showed decreased vacuolar P content. Moreover, the vacuolar P content of the *OsVPE1 OsVPE2* double mutant was higher than that of the wild types. Therefore, it is important to understand P homeostasis to enhance PUE in plant species grown in podzolic soils. Vacuole P could serve an important source to enhance IPUE particularly during P remobilization under P starvation conditions.

### Alternative Biochemical Pathways

Metabolic redundancy could help plants to respond quickly to an unfavorable environment (Plaxton and Tran, 2011). Induction of P-independent or inorganic pyrophosphate (PPi)-dependent glycolytic metabolic reactions might enhance PUE by P recycling and reduction of cellular ATP pool consumption by utilizing alternative P pools under P starvation conditions (Wang et al., 2010). During long-term P stress, a marked decline in nucleoside pools such as ATP and ADP was observed because of cytoplasmic P exhaustion (Plaxton and Podestá, 2006). P-dependent cytosolic glycolysis and the phosphorylating pathway of the mitochondrial electron transport chain could be inhibited. Therefore, alternative reactions of cytosolic glycolysis contribute to maintaining carbon flux to generate energy (Plaxton and Tran, 2011). Ppi is a by-product of various biosynthesis reactions, such as polymerization reactions during nucleic acid and protein, polysaccharide, and macromolecule synthesis, with a concentration of up to 0.5 mM in the cytosol. Unlike animals, plant cells lack soluble Ppiase. The level of Ppi appears to be unaffected compared to adenine nucleotide and uridine nucleotide energy donor systems and shows the importance of Ppi as an initiative energy donor in P-deficient cytosol cells (Theodorou and Plaxton, 1993).

Some energy-poor anaerobic microorganisms use Ppi-phosphofructokinase (Ppi-PFK) instead of ATP-phosphofructokinase (ATP-PFK) to convert Ppi and Fru-6-P into Fru-1,6-P_2_, such as *Priopionibacterium shermanii* and *Entamoeba histolytica* (Mertens, 1991). Additionally, pyruvate P dikinase (PPDK) could also use Ppi to convert adenosine monophosphate (AMP) and phosphoenolpyruvate (PEP) to pyruvate, ATP, and P for P-starved plants (Tran et al., 2010). Significant upregulation of glycolytic enzyme activities of sucrose synthase, UDP-glcpyrophosphorylase, Ppi-PFK, PPDK, and tonoplast Ppi-dependent proton pump (H^+^-Ppiase) has been confirmed during P-starvation in plants (Theodorou and Plaxton, 1994; Palma et al., 2000; Nasr Esfahani et al., 2016). Ppi-dependent phosphorylation of Fru-6-P is catalyzed by Ppi-PFK, which appears to be an adaptive enzyme for coping with P deficiency (Theodorou et al., 1992). Overexpression of H^+^-Ppiase in transgenic *Arabidopsis*, tomato, and rice showed better performance in limited P conditions (Yang et al., 2007). The use of Ppi rather than nucleoside Ps such as ATP and ADP could confer a significant bioenergetic advantage to plants subjected to environmental stresses like in podzolic soils.

## PHE, PAE, AND IPUE: Potential Strategies to Enhance Pue in Podzolic Soils

From the above review, it is evident that seed phytate-P hydrolysis efficiency (PHE), PAE, and IPUE could be fundamental targets to enhance the overall plant PUE during plant growth and harvest. However, it is important to understand which one is more critical considering all available options right from seed phytate hydrolysis, external P uptake, to efficient internal P utilization to attain desirable harvest or produce (Wang et al., 2010; Veneklaas et al., 2012; van de Wiel et al., 2016). For instance, in podzolic soils, the initial seed P stock could play an important role in sustaining early seedling growth and successful establishment for an extended growth period (Figure 3). Boreal climate conditions during early growth stages may restrict root architecture and root morphological traits, seedling establishment, and, finally, biomass accumulation in young seedlings, which could enhance P efflux from seeds, resulting in P losses to the environment in podzolic soils. However, agronomic interventions such as plastic mulching and changing seeding time or depth could provide more crop heat units during early growth stages (Kwabiah et al., 2003; Kwabiah, 2005) and enhance PHE and translocation to developing seedling sinks. After seedling establishment, P uptake is more critical compared to internal P utilization to support plant growth. Organic P sources from soil amendments could provide a better solution to enhance PAE through improved soil microbial communities and enhanced acid phosphatase activities and availabilities of P in podzolic soils (Ali et al., 2019b). Biochar, a recalcitrant carbon, could be an important soil amendment to improve soil physicochemical properties, reduce carbon footprints, and enhance crop productivity in podzolic soils (Ahmed et al., 2016; Altdorff et al., 2018; Ashiq et al., 2020). Literature review and meta-analysis studies reported a wide range of biochar effects, including positive, negative, and neutral, on physicochemical and biological soil fertility and plant responses involving multiple complex mechanisms (Spokas et al., 2012; Ding et al., 2016; Joseph et al., 2021). Effects of biochar application on boreal podzolic soils are still in the initial stages (Abedin and Unc, 2020; Ashiq et al., 2020; Abedin et al., 2021). Only a few studies have documented the superior performance of biochar in improving the fertility of acidic and course soils *via* different mechanisms. The meta-analysis of Glaser and Lehr (2019) calculated an average of 4.6-fold increase in P availability for all soil groups amended with biochar and fertilizers; especially, acidic soils (with pH <6.5) had a 5.1-fold increase in P availability compared to neutral and alkaline soils. The increased P availability in soil groups that received 5–20 Mg biochar ha^−1^ might partly explain the reported crop yield increase by 12–40% (Joseph et al., 2021). P availability and use efficiency could be related to several soil properties improved by biochar addition; one of them is increased (75%) mycorrhizal colonization of crop root compared to 20% for fertilizer alone (Blackwell et al., 2015). Chemical reactions such as ion exchange, redox, adsorption, and desorption activities on the surface of biochar and its liming effect contribute to P availability and use efficiency (Blackwell et al., 2015; Glaser and Lehr, 2019).

Manske et al. (2001) reported that in spring wheat, PUE was determined by PAE with restricted P availability in the growth environment in both acidic and alkaline soils. Low P availability in podzolic soils and shallow nature might reduce overall PUE in growing plants under field conditions particularly from early seedling growth because of low crop heat units. Similarly, the importance of PAE has been highlighted in common bean (Beebe et al., 2006), soybean (Zhao et al., 2004), and wheat (El Mazlouzi et al., 2020). Fujita et al. (2004) and Zhang et al. (2009) suggested that enhancing PAE could be one of the main strategies to enhance PUE in P-deficient soils. This could be achieved through the selection of crop species with better root architecture, more root hair formation, proteoid roots, root exudates, root symbiosis, and phosphate transporters to acquire unevenly distributed P in podzolic soils of boreal agroecosystems. However, in P-adequate production systems, higher IPUE could provide more benefits to improve PUE than PAE. Balemi and Schenk (2009a,b) reported variations in PAE in different potato cultivars, whereas no difference in PUE was observed. Therefore, such reports support the idea that PAE and PUE are mainly controlled by the genetic potential of plants under given growth conditions (Clark and Duncan, 1991; Shenoy and Kalagudi, 2005). For instance, maize plants showed higher PUE associated with PAE rather than IPUE under low P conditions (Corrales et al., 2007). El Mazlouzi et al. (2020) reported higher P translocation of external P to wheat grains under P deficient conditions. Therefore, selecting crop genotypes with better genetic makeup, uptake, and internal utilization efficiency (George and Richardson, 2008; Zhang et al., 2009) could provide a better solution to enhance PUE in crops and at cropping systems level in podzolic soils. Modern tools such as gene- editing could be valuable for precise alteration in genes to improve the genetics of crop plants for PUE.

## Summary

Phosphorus-efficient plants may play a major role in increasing crop yields in podzolic soils because of low soil pH and non-availability of P, rapidly decreasing P resources, and growing environmental concerns. Also, podzolic soils have high P fixation capacities due to Al^3+^ and Fe^2+^, which is a big challenge in enhancing PAE. Literature-reported improvements in PUE are often attributed more to PAE under limited P supply and to IPUE under high P conditions. Therefore, improvement of both PAE and IPUE in a given species in podzolic soils seems to be the appropriate breeding approach along with enhanced seed P reserves that can support good seedling stand. However, conventional breeding strategies are mainly employed to achieve higher biomass or yield in a high-input environment, such as super-hybrid varieties, which require more P fertilizer than other strains to achieve an economically optimum yield. This could enhance financial and environmental burdens. Future further research needs to improve the understanding of podzolic soil amendments, effects of amendments on soil physicochemical properties, soil microbial communities, enzyme activities, and P availability under acidic soil conditions. Accumulation of P in acidic soils due to heavy manure/fertilizer application may cause P toxicity to crop plants in different cropping systems, and this needs to be investigated to assess the effects of excessive P loading to leaves and grains, and the possible deleterious effects on plant growth and development. Furthermore, assessing the organic acid profile of different crop species grown on podzolic soils would enhance the understanding of PUE. Additionally, it is important to investigate the role of P transporters and internal P redistribution and/or any specific plant part that contributes significant P loading to grains and that could be a potentially powerful strategy for increasing P efficiency in modern crops grown in intensive cropping systems. New genetic strategies could also be used to sequester the unavailable P from soil through active and efficient P transporters, and reutilized in P-deficient situations.

## Author Contributions

MN and MC conceived the concept. MN, JW, HG, AK, and SS wrote the first version. MC, AM, and JS provided critical comments. All authors contributed in the final article and approved the submission.

## Conflict of Interest

The authors declare that the research was conducted in the absence of any commercial or financial relationships that could be construed as a potential conflict of interest.

## Publisher's Note

All claims expressed in this article are solely those of the authors and do not necessarily represent those of their affiliated organizations, or those of the publisher, the editors and the reviewers. Any product that may be evaluated in this article, or claim that may be made by its manufacturer, is not guaranteed or endorsed by the publisher.
